# Pleiotropic Effect of *GNP1* Underlying Grain Number per Panicle on Sink, Source and Flow in Rice

**DOI:** 10.3389/fpls.2020.00933

**Published:** 2020-06-19

**Authors:** Laiyuan Zhai, Feng Wang, An Yan, Chengwei Liang, Shu Wang, Yun Wang, Jianlong Xu

**Affiliations:** ^1^Rice Research Institute, Shenyang Agricultural University, Shenyang, China; ^2^Institute of Crop Sciences, National Key Facility for Crop Gene Resources and Genetic Improvement, Chinese Academy of Agricultural Sciences, Beijing, China

**Keywords:** near-isogenic lines, *GNP1*, grain yield, sink–source–flow relationships, grain number per panicle

## Abstract

Rice yield potential is largely determined by the balance among source capacity, sink strength, and flow fluency. Our previous study indicated that the gene *GNP1* encoding gibberellin biosynthesis gene *GA20ox1* affects grain number per panicle (GNP) in rice, thus resulting in increase of grain yield. To clarify *GNP1* effect on sink, source and flow in regulating rice grain yield, we compared Lemont, a *japonica* (*geng*) cultivar, with its near-isogenic line (NIL-*GNP1*^TQ^) in Lemont background with introgression of the allele at *GNP1* from Teqing, a high-yielding *indica* (*xian*) cultivar. NIL-*GNP1*^TQ^ exhibited averagely 32.8% more GNP than Lemont with the compensation by reduced seed setting rate, panicle number and single-grain weight. However, NIL-*GNP1*^TQ^ still produced averagely 7.2% higher grain yield than Lemont in two years, mainly attributed to significantly more filled grain number per panicle, and greater vascular system contributing to photoassimilates transport to spikelets. The significantly decreased grain weight of superior spikelets (SS) in NIL-*GNP1*^TQ^ was ascribed to a significant decrease of grain size while the significantly decreased grain weight of inferior spikelets (IS) ascribed to both grain size and poor grain-filling as compared with Lemont. The low activities of key enzymes of carbon metabolism might account for the poor grain-filling in IS, which resulted in more unfilled grains or small grain bulk density in NIL-*GNP1*^TQ^. In addition, low seed setting rate and grain weight of IS in NIL-*GNP1*^TQ^ might be partially resulted from significantly lower carbohydrate accumulation in culms and leaf sheath before heading compared with Lemont. Our results indicated that significantly increased GNP from introgression of *GNP1*^TQ^ into Lemont did not highly significantly improve grain yield of NIL-*GNP1*^TQ^ as expected, due primarily to significant low sink activities in IS and possible insufficient source supply which didn’t fully meet the increased sink capacity. The results provided useful information for improving rice yield potential through reasonably introgressing or pyramiding the favorable alleles underlying source-related or panicle number traits by marker-assisted selection.

## Introduction

Rice (*Oryza sativa* L.), one of the most important cereal crops and a staple food, feeds over 60% of China’s population. Owing to continuous increasing population, reducing cultivated area, and severe climate environment, a 40% increase in rice production will be necessary during the next 40 years to meet the demands of global food consumption (Murchie et al., 2009). Rice breeding experience indicates that good balance of source, sink and translocation capacity (i.e. flow) of assimilates, namely strength sink, strong source and flow fluency is a prerequisite for high yield potential (Peng et al., 2000). Source is defined as tissues producing photosynthesis such as the upper three leaves, especially the flag leaf, sheath, and so on. Sink is defined as tissues which absorb and utilize photosynthesis, with spikelets being the major primary sink for rice (Li et al., 2018b). The vascular bundle as flow is the transport system that interconnects all parts of the crops, extending from the stem into leaves, and down into the root system, and it determines how crops efficiently deliver photosynthetic products, mineral nutrients and water from source to sink (Housley and Peterson, 1982). Enormous efforts have been made to clarify the physiological mechanism of the relationship among sink size, source capacity and flow transport for improved rice yield potential (Ying et al., 1998; Peng et al., 2000). However, it is difficult to accurately elucidate the physiological function on relationship of sink size, source capacity and flow fluency in previous comparisons at cultivar level due to the major effect of genetic backgrounds of the examined cultivars.

In recent years, several QTL related to source, sink, and flow have been identified and cloned from natural variations, such as sink-related genes *Gn1a* (Ashikari et al., 2005), *DEP1* (Huang et al., 2009), *IPA1* (Jiao et al., 2010), *SPIKE* (Fujita et al., 2013), *GNP1* (Wu et al., 2016). Unfortunately, these genes have not been effectively utilized in modern crop breeding (Li et al., 2018b), with the exception of a limited number of genes such as *IPA1* (Jiao et al., 2010) and *DEP1* (Huang et al., 2009). Ohsumi et al. (2011) reported that NILs carrying superior *Gn1a* and *APO1* alleles did not significantly increase grain yield in the *geng* genetic background because of lack of sufficient substrate supply to fully meet the increased sink demand, illustrating the importance of the harmony between sink size, and source capacity for achieving high rice yield.

The QTL *GNP1* encodes gibberellin biosynthesis gene *GA20ox1*, which increases GNP and yield by increasing cytokinin activity in rice panicle meristems (Wu et al., 2016). In addition, a previous study reported that rice plant statue (plant height) is regulated by *OsGA20ox1* (Oikawa et al., 2004). So, the *GNP1* has pleiotropic effect at least on grain number per panicle (GNP), plant height and flag leaf size (unpublished data). We previously developed a near-isogenic line, NIL-*GNP1*^TQ^ in Lemont genetic background with a unique chromosomal region of 66.1 kb containing *GNP1* locus from the Teqing (Wu et al., 2016). It has been found that NIL-*GNP1*^TQ^ significantly increased GNP by 31–54%, finally resulting in an increase of grain yield of 5.7–9.6% compared with its isogenic control, Lemont, across different environments (Wu et al., 2016). Up to now, it is unclear about physiological mechanism of the superior *GNP1* allele on increase in grain yield. Therefore, evaluating characteristics of sink strength, source capacity, flow fluency, and their interactions would enhance our understanding of the physiological function of *GNP1* gene on regulation of grain yield.

In this study, we compared physiological and yield-related traits, including sink strength such as GNP, grain weight, grain-filling rate, sucrose content, starch content, and activities of the main four key enzymes involved in sucrose-to-starch metabolism of superior spikelets (SS) and inferior spikelets (IS), and source capacity such as photosynthetic rates, chlorophyll content and non-structural carbohydrate (NSC) concentrations in culms and leaf sheaths between NIL-*GNP1*^TQ^ and Lemont. Flow related traits, such as vascular bundle size and number in stem and branches were also measured and compared. Based on these, we will elucidate how the *GNP1* gene to improve grain yield by adjusting the sink–source–flow balance.

## Materials and Methods

### Materials and Field Experiments

Field experiments were performed in the Changping Station (40.2°N; 116.2°E) of the Institute of Crop Sciences, Chinese Academy of Agricultural Sciences in Beijing in 2017 and 2018. Lemont and its isogenic line, NIL-*GNP1*^TQ^ in Lemont background with the introgression allele of Teqing at *GNP1* were used as materials and sowed on 25th April, and a total of 340 plants with 30-day seedlings were transplanted in a plot with a single plant per hill and 17 rows and 20 plants each row at a spacing of 17 cm between hills and 25 cm between rows. Materials were arranged in a randomized block design with three replicates. In the field, 200 kg N ha^−1^ in the form of urea was applied (50% used as basal, 30% applied in 7 days after transplanting, and the remaining 20% applied at the panicle initiation). Phosphorus (90 kg ha^−1^ of P_2_O_5_ in the form of calcium superphosphate) and potassium (90 kg ha^−1^ of K_2_O in the form of potassium chloride) fertilizers was applied as basal before transplanting. The field was irrigated after transplanting with a depth of 3–5 cm until the tillering stage, drained at the maximum tillering stage to control unproductive tillers, and irrigated again at the booting stage with the water layer of 3–5 cm until the heading stage, then performed wetting–drying alternation irrigation during grain filling duration, and finally drained a week before maturity. Field managements followed the local farmers’ practices. Weeds were controlled by a combination of chemical and manual methods. The paddy soil field before the experiment had pH 5.97, organic matter of 10.9 g kg^−1^, available N of 41.7 mg kg^−1^, available P of 116.5 mg kg^−1^, and available K of 98.8 mg kg^−1^.

### Evaluation of Yield and Yield Components

Heading date was recorded when 50% plants emerged panicle in each plot of each genotype. At the full heading stage, plant height was measured on the main stems of eight uniform plants in each plot. At maturity, whole plot was harvested for yield measurement based on a 14% moisture content after air-dried. Above eight plants in each plot were sampled and dried in an oven at 70°C for 5 d for trait investigation including panicle number per plant, panicle length, filled grain per panicle, GNP, thousand grain weight, number of primary rachis branches, number of secondary rachis branches, grain length and grain width. Based on spikelet position on the panicle, the spikelets were separated into four parts, namely on the primary rachis branches (PRB) in the upper, PRB in the lower, secondary rachis branches (SRB) in the upper, and SRB in the lower parts of the panicle. The seed setting rate was calculated by the ratio of fully mature grain number to the total spikelet number. The single-grain weight was determined for the mature grains. Fifteen fully mature grains were randomly selected from above eight plants to measure the grain length and grain width using a Vernier caliper. The derived trait, grain bulk density was calculated as the ratio of grain weight to grain volume.

### Leaf Photosynthetic Measurement

Net photosynthetic rates of rice flag leaves were measured at a photosynthetic photon flux density of 1,500 μmol m^−2^ s^−1^ and a CO_2_ concentration of 350 μmol/mol using a portable system for photosynthesis measurements (LI-6400, LI-COR, Lincoln, NE, USA). The measurements were taken during 9:00–11:00 AM on cloud-free weather in full heading stage. The air temperature of leaf chamber was 28 °C, and the vapor-pressure deficit at the leaf surface was 1.1 kPa. Sixteen flag leaves were selected on the main stems from 16 uniform plants (one flag leaf per plant) in each plot, eight of which were first used for determining the photosynthetic rates in the field, then collected for testing leaf nitrogen content, and the remaining eight flag leaves were used for measuring leaf chlorophyll content for Lemont and NIL-*GNP1*^TQ^. Leaf N content was quantified with an NC analyzer for whole flag leaf. The upper, middle, and lower parts of each selected flag leaf were sampled and mixed for chlorophyll content determination. Leaf chlorophyll content was extracted with ethanol, and measured using spectrophotometer.

### Observation of the Cross-Section of Flag Leaves and Stems

At full heading stage, five flag leaf blades on the main stems from five uniform plants (one leaf blade per plant) selected in the middle of each plot were sampled for observation of the cross-section. The central sections of flag leaf blades were sampled, fixed in formalin-acetic-alcohol (FAA) fixative solution, and embedded in paraffin wax. Transverse sections of leaves with 7 mm thick were cut on a rotary microtome (HM340E, Germany) and stained safranin O and fast green. Leaf mesophyll anatomy at the second large vascular bundles from the midrib was observed and measured under a fluorescence microscope (ZEISS Axio Observer A1, Germany). To measure the stomatal density and stomatal size, we observed the adaxial (upper leaf) and abaxial (lower leaf) surfaces at the middle part of the flag leaf under a scanning electron microscope (SEM) (Hitach TM3030, Japan).

At full heading stage, five main stems from five uniform plants (one stem per plant) were randomly selected in each plot for determination of vascular bundles related traits in peduncle, second node and branches. The transverse section of stem was uniformly made at 2 cm above the peduncle (neck of panicle) and second nodes and kept in FAA fixative solution. And the transverse sections of PRB both in the upper and lower panicle, and SRB in the upper and lower panicle were sampled and fixed in FAA fixative solution. These samples were embedded in paraffin wax, cut 7 mm thick on a rotary microtome (HM340E, Germany) and stained safranin O and fast green. The vascular bundles related traits in peduncle, second node and branches were observed and measured using a microscopy (ZEISS AXIO, Germany).

### Determination of Dry Matter Accumulation and Transportation

Five plants with average tillering number of each plot were harvested at heading and maturity stages in 2017 and 2018. Plant samples were separated into green leaves, stems, senescent organs, and panicles, and their dry weights were determined after oven-drying at 105 °C for 30 min and then at 85 °C to constant weight to calculate total biomass. The oven-dried samples of stem, which included culms and leaf sheaths, were used for determination of non-structural carbohydrate (NSC) concentrations (mg g^−1^ dry weight) according to the method reported previously by Fu et al. (2011). The total mass of NSC stored in stems (g plant^−1^), apparent transferred mass of NSC from stems to grains during grain filling (g plant^−1^), and apparent ratio of transferred NSC from stems to grains (%) were calculated according to the method of Li et al. (2018a). The total green leaf area of each plant was measured using a leaf area meter (LI-300, Li-Cor Inc., Lincoln, NE, USA) in 2018. The leaf area index (LAI) was calculated by the ratio of the total green leaf area of each plant to the area of the ground covered (17 cm × 25 cm). We also measured the length and width of the flag and second leaves on the main stems in 2017 and 2018.

### Determination of Grain-Filling Rate

A total of about 250 panicles heading on the same day were chosen and tagged for each plot of each genotype. Twenty tagged panicles from each plot were sampled every 3 days intervals from 0 to 9 day post anthesis (DPA, the day was accounted from the first day of anthesis), and every 5 days intervals from 9 DPA to maturity. SS (flowering on the first 2 d within a panicle) and IS (flowering on the last 2 d within a panicle) were separated from the sampled panicles, containing about 150 spikelets at each sampling time as described previously by (Ishimaru et al. 2005). Sampled spikelets were dried at 70 °C to constant weight, dehulled, and used to measurement of grain dry weight, sucrose and starch contents. Determination of sucrose content in the spikelets was conducted following the modified method of Yoshida et al. (1976). The starch content was determined as described by Buysse and Merckx (1993). The processes of grain filling were fitted by Richards’ growth equation (Richards, 1959):

$$W=\frac{A}{{\left(1+Be^{-kt}\right)}^{1/N}}$$

### Assay of Enzyme Activity

The method for preparation of enzyme extracts was modified from Nakamura et al. (1989). Briefly, 45 dehulled and frozen grains were homogenized with a pestle in a pre-cooled mortar containing 5 ml frozen extraction medium (100 mmol/L HEPES-NaOH, pH = 7.5, 2 mmol/L EDTA, 50 mmol/L 2-mercaptoenthanol, 12.5% (v/v) glycerol). After being filtered through four layers of cheesecloth, the homogenate was centrifuged at 12,000×*g* for 10 min at 4 °C and the supernatant was directly used for assay of activities of sucrose synthase (SuSase), starch synthase (StSase), starch branching enzyme (SBE), and ADP-glucose pyrophosphorylase (AGPase). Above process was conducted at 0–4°C.

SuSase was assayed in the cleavage direction and analyzed as described by Ranwala and Miller (1998). StSase activity was determined according to the method of Schaffer and Petreikov (1997). Activities of AGPase and SBE were determined according to the method described by Nakamura et al. (1989).

### Statistical Analysis

Statistical analyses were performed using two-way analysis of variance (ANOVA). Genotype, year, and genotype-by-year interactions were treated as fixed effects. Then, the *F*-test was used to test any difference between the two genotypes. Student’s *t*-test (two-tailed) was used to determine exact *P* values. All the analyses were performed in the R software (R Core Team, 2015).

## Results

### Performance of Yield and its Related Traits

**Figure 1:**
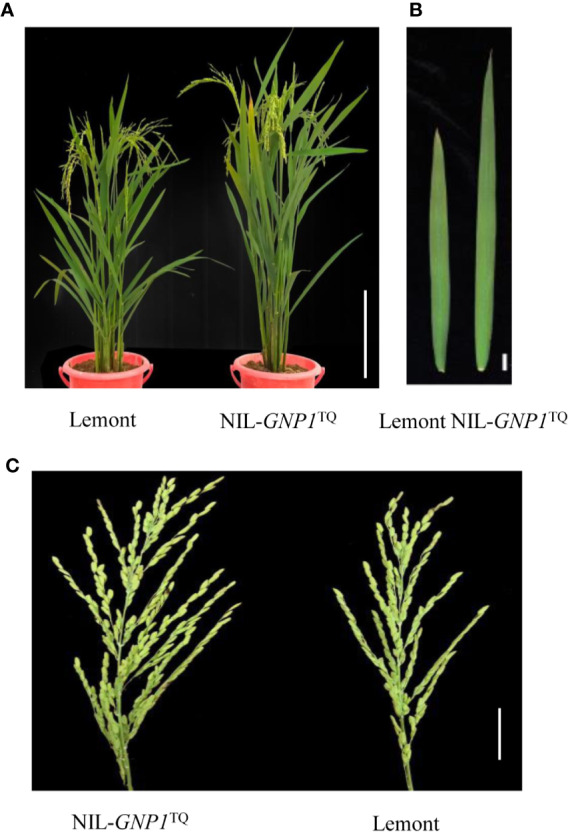
Performance of Lemont and NIL-*GNP1*^TQ^ under natural conditions. **(A)** Gross morphology of Lemont (left) and NIL-*GNP1*^TQ^ (right) (Bar = 40 cm). **(B)** Flag leaf morphology of Lemont (left) and NIL-*GNP1*^TQ^ (right) (Bar = 2 cm). **(C)** Panicle morphology of NIL-*GNP1*^TQ^ (left) and Lemont (right) (Bar = 5 cm).

The day to heading of Lemont was one and two days earlier than that of NIL-*GNP1*^TQ^ in 2017 and 2018, respectively. The GNP of NIL-*GNP1*^TQ^ exhibited averagely 32.8% more than that of Lemont, with much higher (43.5%) number on secondary rachis branches (**Table 1**). In addition, NIL-*GNP1*^TQ^ showed significant increases in plant height (+11%), filled grain number per panicle (+29.9%), and panicle length (+9.6%) but significant decreases in productive panicle number per plant (−8.7%), 1,000-grain weight (−9.5%) and grain length (−2.2%) than Lemont in 2017 and 2018 (**Table 1**; **Figure 1**). Finally, the NIL-*GNP1*^TQ^ produced averagely 7.2% higher grain yield than Lemont across the two years. These results suggested that the *GNP1*^TQ^ is of pleiotropy and has potential value in high yield rice breeding.

**Table 1:** Comparison of yield related traits between Lemont and NIL-*GNP1*^TQ^.

Trait	Lemont^a^	NIL-*GNP1*^TQ^	Analysis of variance^b^		
			Year	Genotype	Year × Genotype
Plant height (cm)	82.9	92.0*	***	***	ns
Productive panicle number per plant	10.3	9.4**	*	**	ns
Filled grain per panicle	134.1	174.2***	*	**	ns
Grain number per panicle	157.1	208.6**	**	***	ns
Primary branch number	11.9	11.9	ns	ns	ns
Secondary branch number	24.6	35.3***	ns	***	ns
Panicle length (cm)	22.9	25.1**	ns	**	ns
1,000-grain weight (g)	24.3	22.0***	ns	***	ns
Grain length (mm)	9.0	8.8*	**	**	ns
Grain width (mm)	2.5	2.5	*	ns	ns
Grain yield (kg ha^-1^)	5901.5	6325.8	ns	ns	ns

a The *, **, *** indicate significant differences between NIL-GNP1^TQ^ and Lemont at P < 0.05, 0.01, and 0.001, respectively based on Student’s t-test.

b The *, **, *** indicate significant level at P < 0.05, 0.01, and 0.001, respectively, based on analysis of variance; ns indicates non-significance based on analysis of variance.

**Table 2** demonstrated the single-grain weight, seed setting rate and grain size in 2018 at four panicle positions. The grain length and width in the NIL-*GNP1*^TQ^ were significantly shorter than those of Lemont at all positions. The single-grain weight in the NIL-*GNP1*^TQ^ significantly decreased by 6.9, 9.9, 9.5, and 14.2% compared with those of Lemont on PBR and SBR at upper, and PBR and SBR at lower panicle positions, respectively, indicating that the single-grain weight on SBR at lower panicle position was most pronounced decrease in NIL-*GNP1*^TQ^. The seed setting rate on SRB at upper and lower panicle positions in the NIL-*GNP1*^TQ^ significantly decreased by 9.9 and 21.6% compared with those of Lemont, respectively, whereas that on PRB at both panicle positions did not markedly differ between Lemont and NIL-*GNP1*^TQ^ (**Table 2**). NIL-*GNP1*^TQ^ showed significantly decrease grain bulk density on SBR at upper, and PBR and SBR at lower panicle positions compared with Lemont, whereas the grain bulk density on PBR at upper panicle position did not significantly differ between them (**Table 2**).

**Table 2:** Comparison of single-grain weight, size and seed setting rate at different panicle positions between Lemont and NIL-*GNP1*^TQ^ in 2018.

Trait	Genotype	Upper		Lower	
		PRB^a^	SRB	PRB	SRB
Grain length (mm)	Lemont	9.34	9.18	9.30	8.60
	NIL-*GNP1*^TQ^	9.13***	8.82***	9.01***	8.42*
Grain Width (mm)	Lemont	2.47	2.45	2.46	2.38
	NIL-*GNP1*^TQ^	2.44*	2.41*	2.42**	2.33**
Seed setting rate (%)	Lemont	92.2	85.6	91.3	80.9
	NIL-*GNP1*^TQ^	90.3	77.1*	88.3	63.4***
Single-grain weight (mg)	Lemont	24.7	24.2	24.3	21.8
	NIL-*GNP1*^TQ^	23.0***	21.8***	22.0***	18.7***
Grain bulk density	Lemont	1.12	1.12	1.11	1.11
(g mm^-3^)	NIL-*GNP1*^TQ^	1.09	1.08*	1.06**	1.01***

a PRB, primary rachis branches; SRB, secondary rachis branches.

The *, **, *** indicate significant difference between NIL-GNP1^TQ^ and Lemont at P < 0.05, 0.01, and 0.001, respectively.

### Performance of Source-Related Traits

Although widths of the flag and second leaves in NIL-*GNP1*^TQ^ were significantly narrower than those in Lemont, NIL-*GNP1*^TQ^ showed significantly larger areas of flag and second leaves compared with Lemont because the former had significant longer flag and second leaves than the latter (**Table 3**; **Figure 1**). There was no significant difference in LAI at the full heading stage between NIL-*GNP1*^TQ^ and Lemont in 2018 (**Table 3**), partly due to the significantly decreased panicle number in the NIL-*GNP1*^TQ^ (**Table 1**). The chlorophyll contents of flag and second leaves of NIL-*GNP1*^TQ^ were similar to those of Lemont (**Table 3**). However, NIL-*GNP1*^TQ^ exhibited slightly decreased but no significantly differences in leaf N content and photosynthetic rate compared with Lemont (**Table 3**). The NSC concentration of NIL-*GNP1*^TQ^ was significantly lower than that of Lemont at the heading stage (**Table 4**).

**Table 3:** Comparison of leaf morphological traits at the full heading stage between NIL-*GNP1*^TQ^ and Lemont.

Genotype	Length		Width		Area		N content		Chlorophyll		LAI (m^2^ m^−2^)	Photosynthetic rate (μmol m^−2^ s^−1^)
	(cm)		(cm)		(cm^2^)		(g m^−2^)		(mg g^−1^)			
	FL^a^	SL	FL	SL	FL	SL	FL	SL	FL	SL		
Lemont	27.5	37.8	1.9	1.6	39.6	46.2	1.1	1.0	1.2	1.2	4.8	20.8
NIL-*GNP1*^TQ^	35.6**	47.3**	1.8*	1.5**	48.8**	53.7**	1.0	0.9	1.2	1.2	4.6	19.4
Analysis of variance ^**b**^												
Year	*	**	ns	ns	***	**	ns	ns	ns	ns	–	**
Genotype	**	***	*	**	***	***	ns	ns	ns	ns	–	ns
Year × Genotype	ns	ns	ns	ns	ns	ns	ns	ns	ns	ns	–	ns

a FL, flag leaf; SL, second leaf; The *, **, *** indicate significant difference between NIL-GNP1^TQ^ and Lemont at P <0.05, 0.01, and 0.001, respectively, based on Student’s t-test.

b The *, **, *** indicate significant level at P <0.05, 0.01, and 0.001, respectively, based on analysis of variance; ns indicates non-significance based on analysis of variance.

**Table 4:** Comparison of non-structural carbohydrate (NSC) concentration and transport capacity in culms and leaf sheath between NIL-*GNP1*^TQ^ and Lemont.

Genotype	NSC concentration		Total mass of NSC		ATM^a^ (g plant^−1^)	AR^b^ (%)
	(mg g^−1^)		(g plant^−1^)			
	Heading	Maturity	Heading	Maturity		
Lemont	237.3	83.6	4.1	1.4	2.6	64.2
NIL-*GNP1*^TQ^	224.6*	47.9**	4.6*	0.7**	3.9**	84.6**
Analysis of variance **^c^**						
Year	**	***	**	***	***	***
Genotype	**	***	**	***	***	***
Year × Genotype	ns	ns	ns	*	ns	**

^a^ATM, apparent transferred mass of NSC from stems to grains; AR, apparent ratio of transferred NSC from stems to grains.

^b^The *, **, *** indicate significant difference between NIL-GNP1^TQ^ and Lemont at P < 0.05, 0.01, and 0.001, respectively, based on Student’s t-test.

^c^The *, **, *** indicate significant level at P < 0.05, 0.01, and 0.001, respectively, based on analysis of variance; ns indicates non-significance based on analysis of variance.

### Observation of the Cross-Section of Flag Leaves

**Figure 2:**
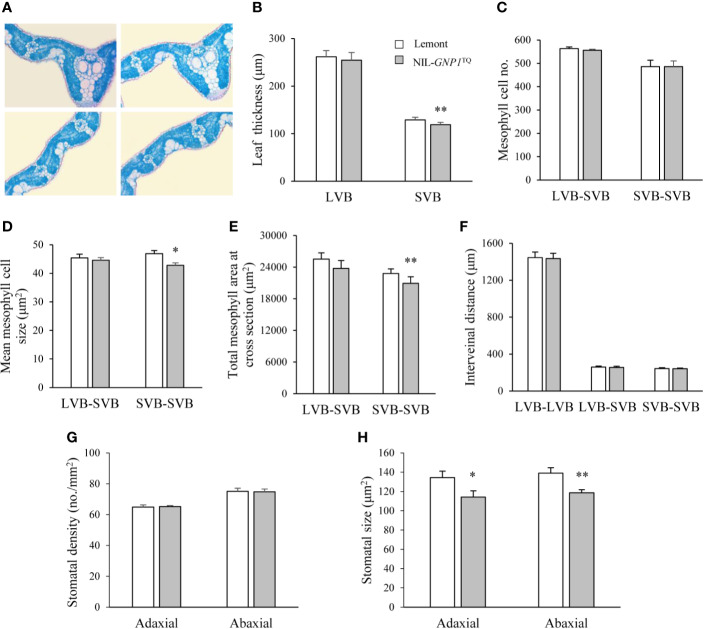
Flag leaf anatomical characteristics in NIL-*GNP1*^TQ^ and Lemont. **(A)** Cross-sections of flag leaves in Lemont (left) and NIL-*GNP1*^TQ^ (right). Comparison of leaf thickness **(B)**, mesophyll cell number **(C)**, mean mesophyll cell size **(D)**, total mesophyll area **(E)**, and interveinal distance **(F)** between LVBs, between LVB and SVB, and between SVBs of flag leaves between NIL-*GNP1*^TQ^ and Lemont. LVB, large vascular bundle; SVB, small vascular bundle. Comparison of stomatal density **(G)** and stomatal size **(H)** on the abaxial and adaxial leaf surfaces between NIL-*GNP1*^TQ^ and Lemont. Error bars indicate standard error (n = 3). The *, ** indicate significant difference between NIL-*GNP1*^TQ^ and Lemont at *P <*0.05 and 0.01, respectively.

We observed cross-sections of the central parts of flag leaves in NIL-*GNP1*^TQ^ and Lemont, and found that NIL-*GNP1*^TQ^ showed significantly less leaf thickness at small vascular bundles (SVB) but no obvious difference at large vascular bundles (LVB) compared with Lemont (**Figures 2A, B**). Although mesophyll cell number between the SVBs was no difference between NIL-*GNP1*^TQ^ and Lemont (**Figure 2C**), NIL-*GNP1*^TQ^ had significantly smaller mesophyll cell size (**Figure 2D**), thus resulting in significantly smaller total mesophyll area between the SVBs in the cross-sections than Lemont (**Figure 2E**). NIL-*GNP1*^TQ^ exhibited similar mesophyll cell number and area between LVB and SVB to Lemont (**Figures 2C–E**). No significant difference was detected in the interveinal distance for different kinds of vascular bundles between NIL-*GNP1*^TQ^ and Lemont (**Figure 2F**). Taken together, above results suggested that smaller mesophyll area gave rise to thinner flag leaf at SVB in NIL-*GNP1*^TQ^.

The comparison revealed that the stomatal densities on abaxial and adaxial leaf surfaces were similar between NIL-*GNP1*^TQ^ and Lemont (**Figure 2G**), whereas the NIL-*GNP1*^TQ^ exhibited significantly smaller stomatal size on the two sides than Lemont (**Figures 2G, H**).

### Biomass Production and Stem Carbohydrates

**Figure 3:**
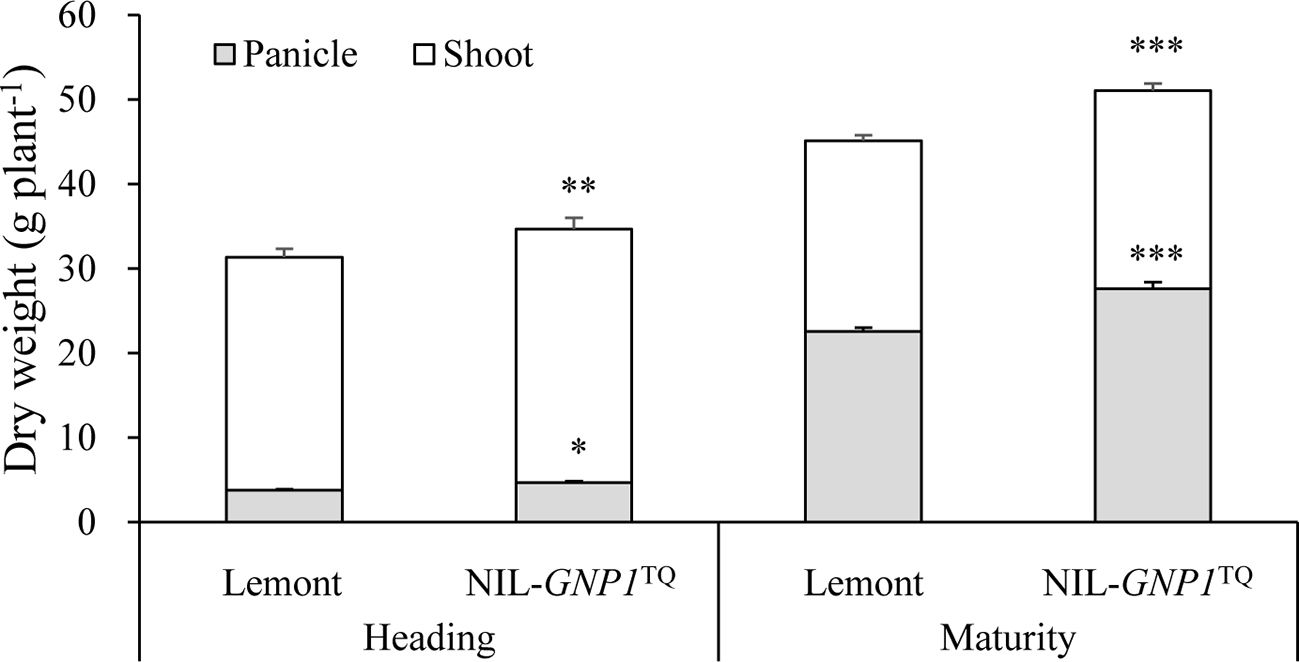
Aboveground biomass accumulation at heading and maturity stages in NIL-*GNP1*^TQ^ and Lemont. The *, **, *** indicate significant difference in panicle weight and total aboveground biomass production between NIL-*GNP1*^TQ^ and Lemont at *P <* 0.05, *P <* 0.01, and *P <*0.001, respectively. Error bars indicate standard error (n = 6).

NIL-*GNP1*^TQ^ exhibited significant increase in total aboveground biomass production at heading and maturity stages compared with Lemont (**Figure 3**). Also, panicle dry matter in NIL-*GNP1*^TQ^ was markedly higher than that of Lemont at heading and maturity stages (**Figure 3**). However, no significant difference in dry weight ratio of panicles to total aboveground biomass was observed between NIL-*GNP1*^TQ^ (average 56.7%) and Lemont (average 55.3%) at maturity.

NIL-*GNP1*^TQ^ accumulated significantly higher total mass of NSC stored in the stem (culms and sheaths) at the heading but significantly lower total mass of NSC at maturity than Lemont (**Table 4**). NIL-*GNP1*^TQ^ showed significantly higher apparent transferred mass of NSC and apparent ratio of transferred NSC from stems to grains than Lemont (**Table 4**).

### Grain Filling Rate and Grain Weight

**Figure 4:**
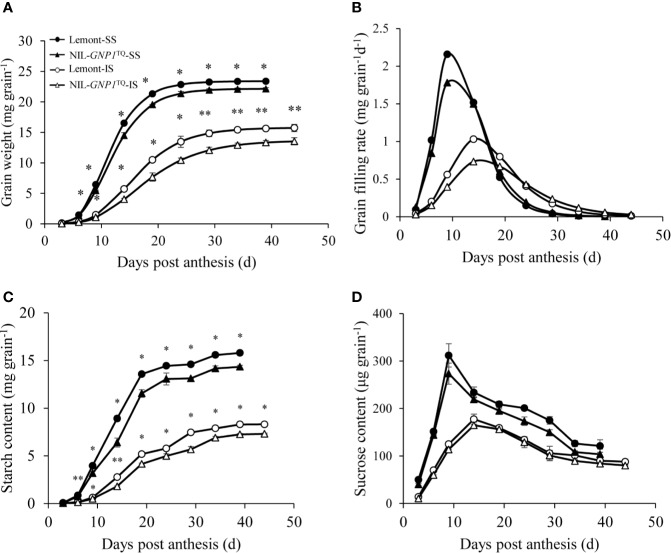
Grain weight **(A)**, grain filling rate **(B)**, starch content **(C)**, and sucrose content **(D)** of superior (SS) and inferior spikelets (IS) in NIL-*GNP1*^TQ^ and Lemont during grain-filling period. The *, ** indicate significant difference between NIL-*GNP1*^TQ^ and Lemont at *P <* 0.05 and *P <* 0.01, respectively. Error bars indicate standard error (n = 3).

The grain filling rate and grain weight of SS were consistently much higher than those of IS in both NIL-*GNP1*^TQ^ and Lemont during the whole grain filling period, and peak value of grain filling rate also occurred earlier in SS than IS (**Figure 4**). Compared with Lemont, NIL-*GNP1*^TQ^ showed lower peak values of the grain filling rate and significantly smaller grain weight during the whole filling period for both SS and IS (**Figures 4A, B**), and its lower grain weight of IS was more aggravated (**Figure 4A**). Specifically, compared with Lemont at maturity stage, the grain weights of SS and IS in NIL-*GNP1*^TQ^ were significantly decreased by 8.6 and 14.1%, respectively. Similar trends were observed in starch content of IS and SS in both NIL-*GNP1*^TQ^ and Lemont. The starch content of IS and SS in NIL-*GNP1*^TQ^ were significantly higher than those of Lemont during the grain-filling period, and the final starch content of SS and IS in NIL-*GNP1*^TQ^ were approximately 5.3 and 14.6% lower than those of Lemont, respectively. It was found that SS had significantly higher sucrose content than IS in both NIL-*GNP1*^TQ^ and Lemont at the early and middle grain-filling stage. NIL-*GNP1*^TQ^ exhibited slightly lower but no significant difference in sucrose contents of SS and IS compared Lemont during grain filling period.

### Activities of Key Enzymes Involved in Metabolism of Sucrose to Starch

**Figure 5:**
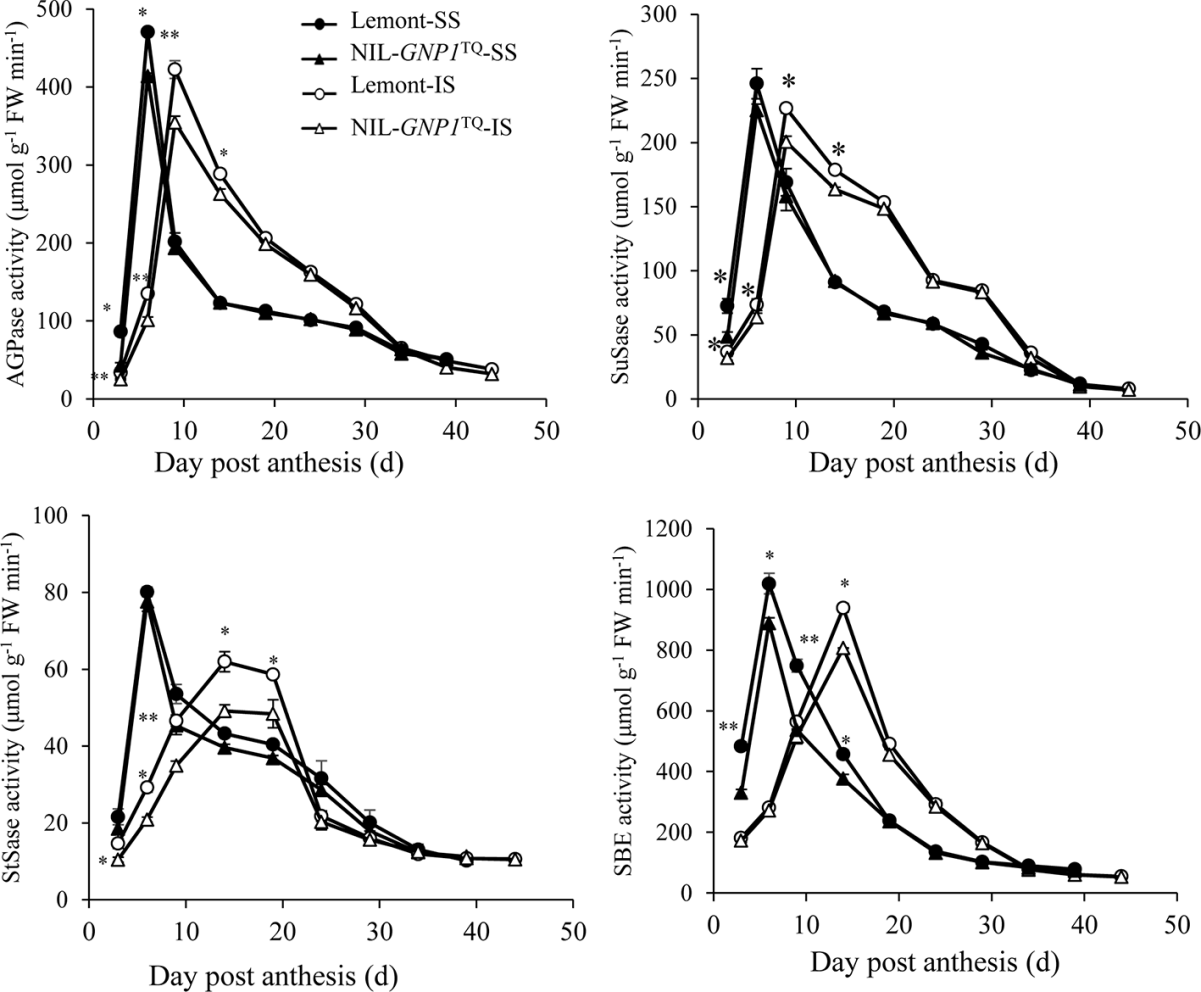
Activities of key enzymes involved in sucrose to starch conversion of superior (SS) and inferior spikelets (IS) in NIL-*GNP1*^TQ^ and Lemont during grain-filling period. Error bars indicate standard error (n = 3). The *, ** indicate significant difference between NIL-*GNP1*^TQ^ and Lemont at *P <* 0.05, and *P <* 0.01, respectively.

In this study, we compared activities of AGPase, SuSase, StSase and SBE, four key enzymes involved in the metabolism of carbohydrates between SS and IS in NIL-*GNP1*^TQ^ and Lemont. The results demonstrated that, similar to grain filling changes, AGPase, SuSase, StSase and SBE activities in SS and IS were relatively low at the beginning of the grain filling stage, followed by increase to maximum activity and then a rapid decrease as grain filling processes (**Figure 5**). Peaks of the four enzyme activities were obviously delayed in IS compared with SS, and the peak values of the four enzyme activities were also lower in IS than SS for both NIL-*GNP1*^TQ^ and Lemont (**Figure 5**). Moreover, NIL-*GNP1*^TQ^ showed significantly lower AGPase activity in SS at early grain filling stage (from 3 to 6 DPA), and significantly decreased the enzyme activity in IS at early and middle grain filling stages (from 3 to 15 DPA) than those Lemont (**Figure 5**). The peak values of AGPase activity of SS and IS in NIL-*GNP1*^TQ^ were 11.9 and 16.1% lower than those in Lemont, respectively. NIL-*GNP1*^TQ^ exhibited significantly lower SuSase activity in SS at the initial grain filling stage (3 DPA), and markedly lower in IS at early and middle grain filling stages (from 3 to 15 DPA) than Lemont (**Figure 5**). Compared with Lemont, the peak value of SuSase activity of SS and IS in NIL-*GNP1*^TQ^ significantly decreased by 8.5 and 11.6%, respectively (**Figure 5**). Although the activities of StSase reached their peak values in SS and IS at different times, the overall trends were similar to that of SuSase, except for significant difference in SuSase of SS at 3 DAP between NIL-*GNP1*^TQ^ and Lemont (**Figure 5**). NIL-*GNP1*^TQ^ showed significantly lower SBE activity in SS at the earlier and middle grain filling period (from 3 to 15 DPA), and significantly lower enzyme activity in IS at the middle grain filling stage (at 15 DPA) than Lemont (**Figure 5**).

### Observation of the Cross-Section of Stems and Branches

**Figure 6:**
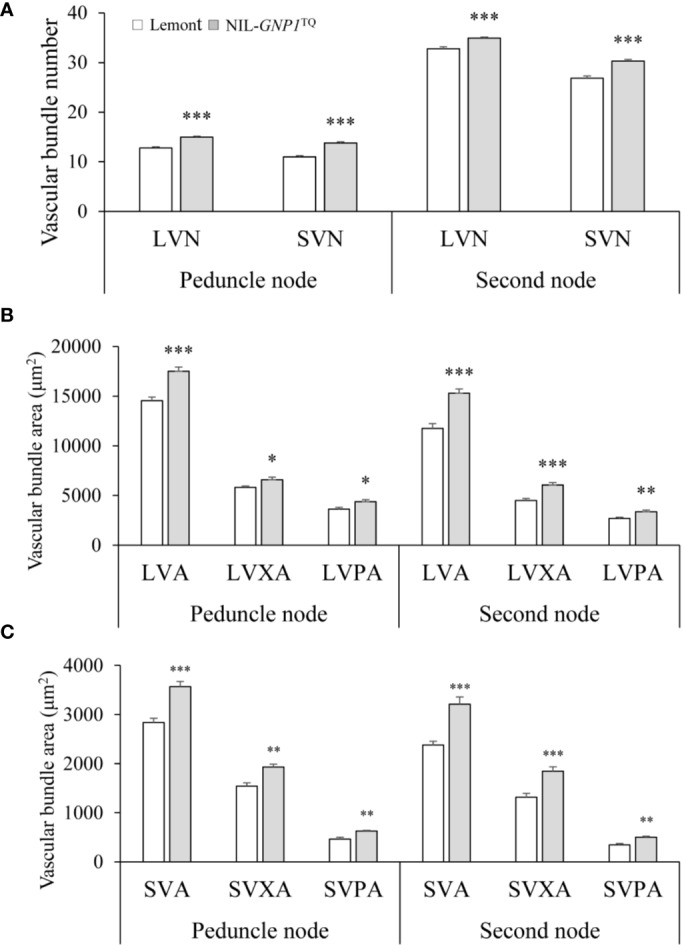
Comparisons of the number of vascular bundles **(A)**, area of large vascular bundles **(B)**, and area of small vascular bundles **(C)** between NIL-*GNP1*^TQ^ and Lemont in peduncle and second node. LVN, the number of large vascular bundle; LVA, the total area of large vascular bundle; LVPA, the phloem area of large vascular bundle; LVXA, the xylem area of large vascular bundle; SVN, the number of small vascular bundle; SVA, the total area of small vascular bundle; SVPA, the phloem area of small vascular bundle; SVXA, the xylem area of small vascular bundle. Error bars indicate standard error (n = 6). The *, **, *** indicate significant difference between NIL-*GNP1*^TQ^ and Lemont at *P <* 0.05, *P <* 0.01, and *P <* 0.001, respectively.

NIL-*GNP1*^TQ^ showed significantly increased number of SVB and LVB in the cross-sections of peduncle and second nodes compared with Lemont (**Figure 6A**). And NIL-*GNP1*^TQ^ also exhibited significantly greater area of SVB and LVB, including total area, phloem area, and xylem area, than Lemont (**Figures 6B, C**), illustrating that NIL-*GNP1*^TQ^ much improved vascular bundle system in stem.

The areas of SVB and LVB in PRB were significantly larger than those in SRB at upper and lower of panicles for NIL-*GNP1*^TQ^ and Lemont. And the areas of SVB and LVB in PRB and SRB of NIL-*GNP1*^TQ^ were similar to those of Lemont at upper and lower of panicle (**Table 5**).

**Table 5:** Comparison of area of vascular bundles at different panicle positions between Lemont and NIL-*GNP1*^TQ^ in 2018.

Trait^a^	Genotype	Upper		Lower	
		PRB^b^	SRB	PRB	SRB
LVA(μm^2^)	Lemont	10,532	4,132	10,009	4,216
	NIL-*GNP1*^TQ^	9,914	4,301	9,944	4,380
LVPA(μm^2^)	Lemont	2,513	1,190	2,595	1,144
	NIL-*GNP1*^TQ^	2,592	1,236	2,455	1,210
LVXA(μm^2^)	Lemont	4,089	1,673	4,016	1,681
	NIL-*GNP1*^TQ^	4,162	1,733	4,033	1,688
SVA(μm^2^)	Lemont	2,649	1,074	2,508	1,080
	NIL-*GNP1*^TQ^	2,791	1,153	2,797	1,140
SVPA(μm^2^)	Lemont	481	242	447	230
	NIL-*GNP1*^TQ^	514	255	502	227
SVXA(μm^2^)	Lemont	917	489	974	530
	NIL-*GNP1*^TQ^	1,051	513	1,074	542

a LVA, the total area of large vascular bundle; LVPA, the phloem area of large vascular bundle; LVXA, the xylem area of large vascular bundle; SVN, the number of small vascular bundle; SVA, the total area of small vascular bundle; SVPA, the phloem area of small vascular bundle; SVXA, the xylem area of small vascular bundle.

b PRB, primary rachis branches; SRB, secondary rachis branches.

## Discussion

We previously developed a near-isogenic line, NIL-*GNP1*^TQ^ in Lemont genetic background with a unique chromosomal segment of 66.1 kb (36,123,921–36,189.989 bp) on chromosome 3, containing the *GNP1* locus from the Teqing (Wu et al., 2016). Based on the Nipponbare reference genome IRGSP 1.0, the region contains 11 annotated genes, including *LOC_Os03g63980* and *LOC_Os03g63990* which are predicted to encode transposon and retrotransposon proteins, respectively; *LOC_Os03g63999*, *LOC_Os03g64010*, *LOC_Os03g64014*, and *LOC_Os03g64020* are expressed protein with unknown function; *LOC_Os03g63940* has CBS domain containing membrane protein with unknown function; *LOC_Os03g64030* and *LOC_Os03g64050* are predicted to encode receptor protein kinase; *LOC_Os03g64070* encode 30S ribosomal protein with unknown function; and *LOC_Os03g63970* (*GNP1*) encodes GA 20-oxidase 1. In the chromosomal region, no other gene except *GNP1* was reported to be associated with GNP or grain yield. Therefore, the variations on physiological and yield-related traits between NIL-*GNP1*^TQ^ and Lemont are mainly attributed to the effect of *GNP1*.

### Effect of *GNP1* on Sink Size

The Teqing allele of *GNP1* significantly increased grain number by an average of 32.8% in the Lemont genetic background (**Table 1**), in agreement with our previous study (Wu et al., 2016). However, NIL-*GNP1*^TQ^ showed improved total sink size (calculated by GNP × panicle number × single-grain weight) of 9.7% on average compared with Lemont. One of the limiting factors for increase of total sink size in NIL-*GNP1*^TQ^ was the decreased panicle number. NIL-*GNP1*^TQ^ exhibited averagely 8.7% fewer productive panicles per plant than Lemont in two years (**Table 1**). Similar to our results, the NIL carrying *qGN4.1* gene, which is identical to *SPIKE* (Fujita et al., 2013), produced 13–44% more spikelet number per panicle but 9–44% fewer productive tillers than their corresponding isogenic control in six different genetic backgrounds (Singh et al., 2018). The negative correlation between grain number and panicle number, which is also known as the concept ‘yield component compensation’, might be partly explained by the developmental trade-off between tiller or panicle number and spikelet formation owing to competition for nitrogen during spikelet differentiation in rice (Grafius, 1978). ‘Yield component compensation’ has been largely responsible for the failure in breeding efforts to improve yield potential through indirect selection of yield components in major cereals (Grafius et al., 1976).

The other factor which limited the increase in sink size in the NIL-*GNP1*^TQ^ was the obvious decrease in single-grain weight. Previous studies demonstrated that NILs carrying grain number genes, such as *APO1* (Terao et al., 2010), *DEP1* (Huang et al., 2009), *SPIKE* (Fujita et al., 2013), *GNP1* (Wu et al., 2016), showed significantly improved grain number per panicle but significantly decreased grain weight in rice. A similar tendency was also found in *GNP1* in this study. The phenomena of low grain weight are common in cultivars with large panicles or extra-heavy types, such as the new plant type (NPT) developed by the International Rice Research Institute (IRRI) (Peng et al., 1999), hybrid rice and super rice or super hybrid rice released in China (Cheng et al., 2007; Peng et al., 2008), which are mainly attributed to poor grain filling of IS and finally produces empty or poor filled grains of rice (Yang and Zhang, 2006; Yang and Zhang, 2010). In this study, we found similar trend that SS exhibited faster grain filling and produced larger and heavier grains than IS in NIL-*GNP1*^TQ^ and Lemont (**Figure 4**). Moreover, due to more grain number NIL-*GNP1*^TQ^ significantly decreased single-grain weight in both SS and IS, especially in IS during the whole grain-filling period as compared with Lemont (**Figure 4**). Grain size and degree of grain filling (grain bulk density) are two parameters, which can determine the grain weight. NIL-*GNP1*^TQ^ showed significantly decreased grain size, and significantly decreased single-grain weight at each spikelet position. And decrease in single-grain weight was most noticeably on SBR at lower panicle position (**Table 2**). NIL-*GNP1*^TQ^ had slightly lower but no significant difference in grain bulk density on PBR at upper panicle position, indicating that the grain bulk density in SS on PBR at upper panicle position of NIL-*GNP1*^TQ^ was similar to that of Lemont. Therefore, we speculated that the final grain weight of SS might be mainly due to significant decrease in grain size in NIL-*GNP1*^TQ^.

Sink strength of rice is determined by sink activity and sink size. Of them, the sink activity is mainly determined by the level of enzyme activity in the sink tissue(s). It has been reported that the poor grain-filling and small grain weight of IS might be mainly attributed to low sink activity, e.g. low activities of key enzymes in carbon metabolism, such as SuSase, StSase, AGPase and SBE (Yang and Zhang, 2010). In this study, the grain bulk density on SBR at lower panicle position of NIL-*GNP1*^TQ^ was remarkably lower than that of Lemont (**Table 2**), most probably resulting from markedly lower activities of AGPase, SuSase, StSase and SBE in IS at early and/or middle grain filling stages. In addition, You et al. (2016) proposed that the slow grain filling and low grain weight of IS were mainly attributed to limitations in the carbohydrate supply. Thus, we inferred that lower final grain weight in IS of NIL-*GNP1*^TQ^ was the result of smaller grain size and lower activities of the four key enzymes involved in the metabolism of carbohydrates. In addition, the poor grain filling and low grain weight of IS in NIL-*GNP1*^TQ^ also might be affected by source supply.

### Effect of *GNP1* on Source Supply

Grain filling in rice is determined by the substrate supply derived from the supply of leaf photosynthesis products after heading, and from carbohydrate stored in the culm and leaf sheaths before heading (Yoshida, 1972). NIL-*GNP1*^TQ^ had significantly larger leaf length and leaf area than Lemont (**Table 3**). The increased leaf area in NIL-*GNP1*^TQ^ compensated for the decreased panicle number, leading to no significantly increase in LAI (**Table 3**). The decreased mesophyll cell area produced thinner leaf thickness at SVB (**Figures 2B–E**) and ultimately led to slightly lower but no significant difference in photosynthesis rate per unit leaf area and leaf N content (**Table 3**) in NIL-*GNP1*^TQ^ as compared with Lemont. Franks and Beerling (2009) demonstrated that increase in total stomatal area potentially lead to improve the maximum stomatal conductance and photosynthesis. The stomatal sizes on abaxial and adaxial leaf surfaces in NIL-*GNP1*^TQ^ were significantly smaller than those of Lemont, whereas NIL-*GNP1*^TQ^ and Lemont displayed similar stomatal density values. The decrease in total stomatal area of NIL-*GNP1*^TQ^ might be associated with slight decrease in photosynthesis rate.

On the other hand, NIL-*GNP1*^TQ^ exhibited significantly lower NSC content in the culm and leaf sheaths than Lemont at heading (**Table 4**). These results showed that source supply to the spikelets might not fully satisfy the increased demand from the greater sink size in NIL-*GNP1*^TQ^. Generally, the photosynthesis assimilate is always preferentially supplied to the SS; therefore the grain filling rate of the IS maybe decrease when photosynthetic products are in short supply (Murty and Murty, 1982). Previous studies demonstrated that Lemont tend to produce more photosynthate than used by grain filling, and the remaining may have deposited in stems and partially contributed to its ratooning ability (Li et al., 1998). Therefore, insufficient source supply probably had little influence on grain filling in SS. However, the poor grain filling and lower grain weight in IS of NIL-*GNP1*^TQ^ might be partially ascribed to less source supply not to fully satisfy the increased sink demand.

### Effect of *GNP1* on Flow Capacity

At heading stage, NIL-*GNP1*^TQ^ exhibited significantly increased total mass of NSC in the culm and leaf sheaths, and significantly increased aboveground biomass accumulation compared with Lemont. It is interesting that NIL-*GNP1*^TQ^ showed significantly lower total mass of NSC in stems at maturity than Lemont; this change in total mass of NSC might be contribute to significantly higher panicle weight per plant in NIL-*GNP1*^TQ^. In other words, NIL-*GNP1*^TQ^ presented a higher apparent transferred mass of NSC from stems to grains than Lemont. In addition, the NIL-*GNP1*^TQ^ exhibited stronger vascular bundles system including significantly more number of vascular bundles and significantly larger area of vascular bundles than Lemont (**Figure 6**), which probably contributed to the improved translocation of carbohydrates from NSC. These results demonstrated NIL-*GNP1*^TQ^ with increased sink size might enhance transformation ability of NSC to some extent, thereby increase grain yield.

### Implications of *GNP1* in Rice Breeding of High Yield Potential

Interaction between QTL/gene and environment has obvious effect on most quantitative traits in rice (Wang et al., 2020). In this study, although Lemont and its isogenic line (NIL-*GNP1*^TQ^) were grown in same location (Beijing), some traits, especially plant height, flag leaf area, and total aboveground biomass production showed statistically significant differences (*P <*0.001) between two years base on analysis of variance (**Tables 1**, **3**, and **4**; **Tables S1** and **S2**), hinting that these traits might be more sensitive to environmental factors. The significant differences in these traits between the two years might be attributed to different environmental conditions in two years. It had more days with higher temperature in 2017 than in 2018 during entire rice growth especially at vegetative growing stage, possibly resulting in significant reduced growth duration, small panicle size and lower aboveground biomass including flag leaf size and plant height of Lemont and NIL-*GNP1*^TQ^ in 2017. Considering *GNP1*^TQ^ is sensitive to environment, attention should be much paid to its application in rice breeding of high yield potential by marker-assisted selection.

Previous studies indicated that the major limiting factor for improved rice yield potential in Lemont is its small sink size rather than source capacity (Li et al., 1998). Our study demonstrated that introgression of the *GNP1* Teqing allele into the Lemont significantly increased averagely 32.8% spikelets number per panicle, ultimately produced averagely 9.7% higher sink size and averagely 7.2% higher yield than Lemont. Yield improvement in NIL-*GNP1*^TQ^ might be explained by significantly increased averagely 29.9% filled grain number per panicle, and improved vascular system contributing to assimilate supply to spikelets. However, the yield advantage (7.2%) in NIL-*GNP1*^TQ^ over Lemont was slightly less than increase in sink size (9.7%) due to the slightly reduction in the seed setting rate. The decrease in seed setting rate in NIL-*GNP1*^TQ^ might be attributed to insufficient source supply resulting in the production of many empty grains. The compensation was taken place in NIL-*GNP1*^TQ^ with increased spikelet number per panicle, fewer productive panicle number and decreased grain weight. The low seed setting rate and single-grain weight of IS in NIL-*GNP1*^TQ^ might be partially attributed to lacks sufficient source supply to fully satisfy the increased sink demand. Ohsumi et al. (2011) reported that introgression of QTL (*SBN1* + *PBN6*) that increase spikelet number per panicle can slightly improve the yield of Sasanishiki because of the slight source surplus, mainly derived from carbohydrates stored in the stem, leading to decrease in seed fertility, panicle number and single-grain weight. These suggests that it is difficult to significantly improve rice yield potential by separately introgressing one or two genes influencing sink size or source supply due to disharmony between sink and source (Jiang et al., 2016). Therefore, it is possible effective to further improve the grain yield of NIL-*GNP1*^TQ^ by reasonably introgressing the multiple favorable alleles of genes underlying source supply-related traits, such as *GPS* controlling photosynthesis rate by regulating carboxylation efficiency (Takai et al., 2013), and *CAR8* for flag leaf nitrogen content, stomatal conductance and photosynthesis in rice (Adachi et al., 2017), or to introgress the superior alleles of genes for increased panicle number, such as *OsMADS57* (Guo et al., 2013) to well coordinate source, sink and flow relationships.

## Conclusions

NIL-*GNP1*^TQ^ exhibited averagely 32.8% more GNP but significantly decreased panicle number, decreased seed setting rate and single-grain weight compared with Lemont. NIL-*GNP1*^TQ^ produced averagely 7.2% higher grain yield than Lemont, mainly due to significantly more filled number per panicle and greater vascular system. The significantly increased GNP derived from introgression of *GNP*^TQ^ allele in Lemont did not highly significantly improve grain yield of NIL-*GNP1*^TQ^ as expected, due primarily to significant low sink activities in IS and possible to insufficient source supply to fully meet the increased sink capacity.

## Data Availability Statement

All datasets presented in this study are included in the article/**Supplementary Material**.

## Author Contributions

YW and JX designed the experiment. LZ, FW, AY, and CL performed all the phenotypic evaluation. LZ and SW performed analysis and interpretation of the data. LZ, YW, and JX drafted the manuscript. YW and JX revised the MS. All authors contributed to the article and approved the submitted version.

## Funding

This work was funded by the National Natural Science Foundation of China (31671602) and the Agricultural Science and Technology Innovation Program and the Cooperation and Innovation Mission (CAAS-ZDXT202001).

## Conflict of Interest

The authors declare that the research was conducted in the absence of any commercial or financial relationships that could be construed as a potential conflict of interest.

## Abbreviations

PRB, primary rachis branches; SRB, secondary rachis branches; GNP, grain number per panicle; QTL, quantitative trait locus/loci; SS, superior spikelets; IS, inferior spikelets; LAI, Leaf area index; NSC, non-structural carbohydrate; SuSase, sucrose synthase; SBE, starch branching enzyme; AGPase, ADP-glucose pyrophosphorylase; SVB, small vascular bundle; LVB, large vascular bundle; DPA, day post anthesis; NIL, near-isogenic lines; FAA, formalin-acetic-alcohol.
