# Supplementary material for: Pleiotropic Effect of GNP1 Underlying Grain Number per Panicle on Sink, Source and Flow in Rice
Source: Front Plant Sci. 2020 Jun 19;11:933. doi: 10.3389/fpls.2020.00933 (PMC7325936; doi:10.3389/fpls.2020.00933)
Supplement: Supplementary file 1 [file Table_1.docx]

**Table S1** Analysis of variance (ANOVA) for yield related traits.

| Stage | Traits | Year | Genotype | Year × Genotype |
| --- | --- | --- | --- | --- |
| Heading | panicle weight | *** | *** | ns |
|  | total aboveground biomass production | *** | *** | ns |
| Maturity | panicle weight | ns | *** | ns |
|  | total aboveground biomass production | *** | *** | ns |

*The *** indicate significant level at P < 0.001 based on analysis of variance; ns indicates non-significance based on analysis of variance.*
