# Supplementary material for: Pleiotropic Effect of GNP1 Underlying Grain Number per Panicle on Sink, Source and Flow in Rice
Source: Front Plant Sci. 2020 Jun 19;11:933. doi: 10.3389/fpls.2020.00933 (PMC7325936; doi:10.3389/fpls.2020.00933)
Supplement: Supplementary file 2 [file Table_2.docx]

**Table S2** Analysis of variance (ANOVA) for vascular bundles-related traits in peduncle and second node

| Traits | Peduncle node | | | Second node | | |
| --- | --- | --- | --- | --- | --- | --- |
|  | Year | Genotype | Year × Genotype | Year | Genotype | Year × Genotype |
| LVN | ns | *** | ns | ns | ** | ns |
| LVA | ns | ** | ns | * | *** | ns |
| LVXA | ns | * | ns | * | *** | ns |
| LVPA | ns | * | ns | ns | ** | ns |
| SVN | ns | *** | ns | ** | *** | ns |
| SVA | ns | *** | ns | ns | ** | ns |
| SVXA | * | *** | ns | ns | ** | ns |
| SVPA | *** | *** | ** | * | *** | ns |

*LVN, the number of large vascular bundle; LVA, the total area of large vascular bundle; LVPA, the phloem area of large vascular bundle; LVXA, the xylem area of large vascular bundle; SVN, the number of small vascular bundle; SVA, the total area of small vascular bundle; SVPA, the phloem area of small vascular bundle; SVXA, the xylem area of small vascular bundle. The *, **, *** indicate significant level at P < 0.05, 0.01, and 0.001, respectively, based on analysis of variance; ns indicates non-significance based on analysis of variance.*
